# Combined Intratympanic and Systemic Steroid Therapy for Poor-Prognosis Sudden Sensorineural Hearing Loss

**Published:** 2013

**Authors:** Shima Arastou, Ardavan Tajedini, Pedram Borghei

**Affiliations:** 1 *Otorhinolaryngology Research Center, Amiralam Hospital, Tehran University of Medical Science, Thran, Iran.*

**Keywords:** Intratympanic Dexamethasone, Sudden deafness, Sudden hearing loss

## Abstract

**Introduction:**

The aim of this study was to evaluate the efficacy of combined intratympanic and systemic steroid therapy compared with systemic steroid therapy alone in idiopathic sudden sensorineural hearing loss (ISSNHL) patients with poor prognostic factors.

**Materials and Methods:**

Seventy-seven patients with sudden sensorineural hearing loss (SSNHL) who had at least one poor prognostic factor (age greater than 40 years, hearing loss more than 70 db, or greater than a 2-week delay between the onset of hearing loss and initiation of therapy) were included in this study. Patients were randomized to the intervention group (combined intratympanic and systemic steroid therapy) or the control group (systemic steroid therapy alone). All patients received oral treatment with systemic prednisolone (1 mg/kg/day for 10 days), acyclovir (2 g/day for 10 days, divided into four doses), triamterene H (daily), and omeprazole (daily, during steroid treatment), and were advised to follow a low salt diet. The intervention group also received intratympanic dexamethasone injections (0.4 ml of 4 mg/ml dexamethasone) two times a week for two consecutive weeks (four injections in total). A significant hearing improvement was defined as at least a 15-db decrease in pure tone average (PTA).

**Results:**

Among all participants,44 patients(57.14%) showed significant improvement in hearing evaluation. More patients showed hearing improvement in the intervention group than in the control group (27 patients (75%) versus 17 patients (41.4%), respectively; P=0.001).

**Conclusion:**

The combination of intratympanic dexamethasone and systemic prednisolone is more effective than systemic prednisolone alone in the treatment of poor-prognosis SSNHL.

## Introduction

 Sudden sensorineural hearing loss (SSNHL), first defined by De Kleyn in 1944(1), is one of the most challenging issues in otolaryngology. Etiological factors can be found in only 10–15% of SSNHL patients, while other patients remain idiopathic (2). Idiopathic SSNHL (ISSNHL) is generally defined as a sensorineural hearing loss of 30 db or more, affecting three or more consecutive audiometric frequencies, with an abrupt onset within 3 days or fewer, without any identifiable cause (‘30/3/3’) (1,3,4). Others define it as a rapid-onset sensorineural hearing loss that develops within 24 h (1). SSNHL affects 5 to 10 individuals per 100,000 population annually (1,2,5,6). The spontaneous recovery rate without treatment ranges from 32–65% (1,2,4), with treatment success ranging from 49–79% (7). 

A significant hearing improvement is defined as at least 15 db decrease in PTA or 20% increase in speech discrimination (SDS) (1,3). According to previous studies (1,8,9,10,11), factors associated with poor prognosis include age greater than 40 years, severe hearing loss, vertigo, high frequency hearing loss, and delays in treatment.

Systemic steroid therapy is currently the mainstay of treatment for ISSNHL (3). Intratympanic steroids are also used for treatment of ISSNHL according to three main protocols: as an initial treatment without systemic steroids, as adjunctive treatment given concomitantly with systemic steroids, and as salvage therapy after failure of systemic steroids (1). Previous studies have shown that intratympanic steroids as initial treatment and salvage treatment are beneficial in the treatment of ISSNHL patients (1); but controversy exists regarding the efficacy of combination therapy with systemic and intratympanic steroids (1).The aim of this study was to evaluate the efficacy of combined intratympanic and systemic steroid therapy compared with systemic steroid therapy alone in ISSNHL patients with poor prognostic factors.

## Materials and Methods

Study participants were chosen among cases of ISSNHL referred to Amiralam Hospital (an ear, nose, and throat (ENT) referral center in Tehran) between June 2008 and November 2009. ISSNHL was defined as rapid-onset sensorineural hearing loss that developed within 24 h, without identifiable cause including retro cochlear disease or trauma (1). Subjects were eligible for inclusion in the study if they had at least one poor prognostic factor: age greater than 40 years, hearing loss more than 70 db, or greater than a 2-week delay between the onset of hearing loss and initiation of therapy. Patients were excluded if they had hypertension, diabetes mellitus, tympanic perforation in the affected ear, history of surgery on the affected ear, bilateral SSNHL, ISSNHL in the hearing ear only, if they were pregnant, or if they received any therapy for SSNHL prior to enrollment in the study. Informed consent was obtained from all participants before entering the study. The study was approved by the local ethics committee of the Department of Otolaryngology in Amiralam Hospital.

At baseline, a standard ENT examination and baseline audiometric evaluation (including PTA, SDS, and acoustic reflex) were performed in all patients. Laboratory studies included blood cell count, coagulation profile, measurement of blood glucose, lipid levels, blood urea nitrogen (BUN), creatinine, erythrocyte sedimentation rate, C-reactive protein (CRP), antinuclear antibody (ANA), rheumatoid factor, syphilis serology (fluorescent treponemal antibody-absorption; FTA Abs), human immunodeficiency virus (HIV) antibody, and urine analysis. Magnetic resonance imaging (MRI) examination of cerebellopontine (CP) angle and internal auditory canal was performed in all patients.

Patients were randomized to the intervention or control groups using a series of computer-generated random numbers. The control group received oral treatment with systemic prednisolone (1 mg/kg/day for 10 days), acyclovir (2 g/day for 10 days, divided in four doses), triamterene H (daily), and omeprazole (daily, during steroid treatment), and were advised to follow a low salt diet.

The intervention group received the same treatment as the control group, in combination with intratympanic dexa- methasone injections (0.4 ml of 4 mg/ml dexamethasone) two times a week for two consecutive weeks (four injections in total). The procedure was performed in the supine position, with the head tilted 45° to the healthy side, under a microscope. After administration of local anesthesia using a lidocaine 10% pump spray, an anterosuperior puncture was made in the tympanic membrane by using a 25-gauge needle and insulin syringe, and the solution was introduced through the needle (11-13). Patients were instructed to avoid swallowing or moving for 20 min after the injections. 

Post-treatment PTA was performed 2 weeks after treatment in both groups (12). The audiologist was blinded to the study group of the patient. PTA was calculated as the average of the thresholds at 0.25, 0.5, 1, 2, and 4 kHz. 

A significant hearing improvement was defined as a decrease of at least 15 db in PTA (1,3,11).

Baseline characteristics of the intervention and control groups are presented as mean (standard deviation) for continuous variables, and the difference between groups was analyzed using Student’s t-test. Categorical variables and the percentage of participants with a significant hearing-improvement outcome were compared using the *X*^2 ^test. The outcome of all participants was analyzed according to the groups to which they were randomized (intention-to-treat analysis). All statistical analysis was performed using Statistical Package for Social Sciences, Version 13.0 (SPSS 13.0, SPSS Inc; Chicago, IL, USA) software.

## Results

Between June 2008 and December 2009, 77 patients with SSNHL who met the inclusion criteria were entered into the study. Patients were randomly divided into two groups, with 41 patients in the control group and 36 in the intervention group. All participants received the therapy to which they were randomized, and all patients completed the therapy. There was no significant difference in the baseline characteristics between the intervention and control groups (Table 1).

**Table 1:** Demographics of SSNHL patients in the present study

	**Intervention Group (n:36)**	**Control** ** Group (n:36)**	**P** ** value**
Age	45.4Y(14.8)^*^	49.17Y(14.4)	0.26
Sex(femaile/male)	11(30%)	10(24%)	0.55
Side(right/left)	19/17	18/23	0.44
Tinnitus	80%	78.10%	0.85
Vertigo	28.6%	34.4%	0.61
Delay to treatment(days)	18.97(23.6)^*^	15.5(22.6)^*^	0.52
Initial PTA	70.7(26.8)^*^	65.9(30.9)^*^	0.47
Poor Prognostic factors			
Age>40	26(72%)	34(82%)	0.26
Hearing loss^1^>70	20(55.6%)	14(34.4%)	0.22
Delay^2^_>_2weeks	15(41.6%)	14(34.4%)	0.50

^1^Initial hearing loss more than 70 db, ^2^Delay from onset of disease to treatment more than 2 weeks  ^*^Values are mean (standard deviation

**Fig 1:**
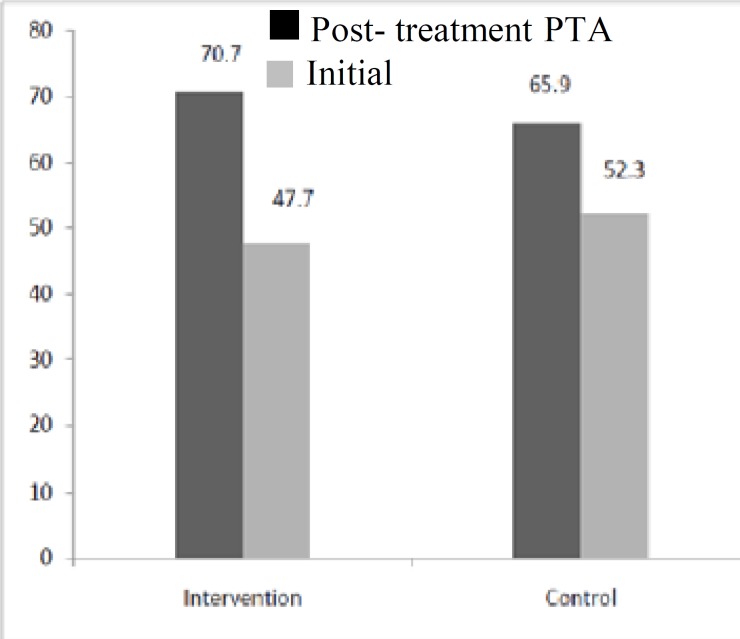
Mean initial and post-treatment PTA (at 250, 500, 1000, 2000, and 4000 Hz) in the intervention and control groups (P= 0.08

**Fig 2:**
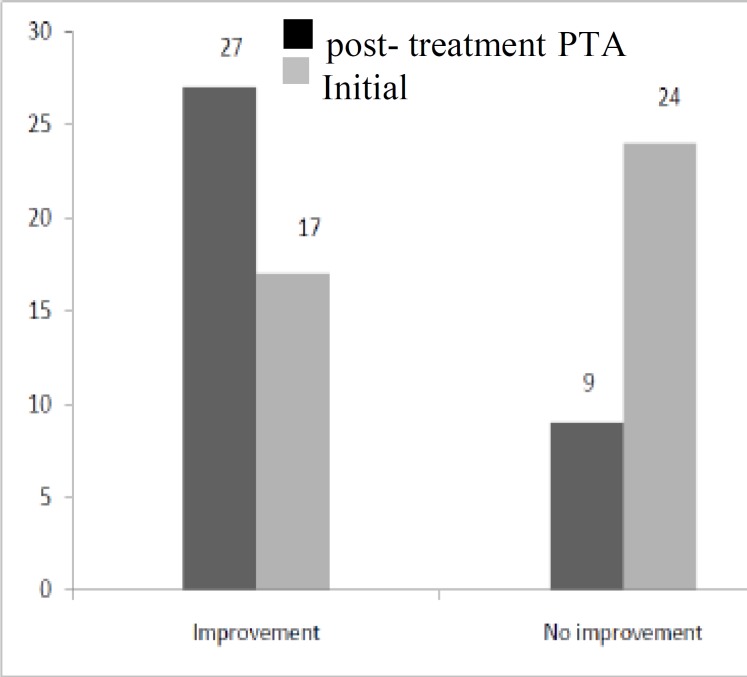
Hearing improvement in intervention and control groups. Improvement is defined by a 15-db decrease in PTA (P=0.001

MRI was performed for all patients, and no neurologic or retrocochlear disorders were revealed. Mean (SE) hearing improvement as measured by PTA was significantly greater in the intervention group than in the control group (22.6 (3.7) versus 13.8 (3.3), respectively; P= 0.08) (Fig 1). Among all participants, 44 patients (57.14%) showed significant improve- ment in hearing evaluation, including 27 (75%) in the intervention group and 17 (41.4%) in the control group (P=0.001) (Fig 2). Two patients (2.6%) developed tympanic perfora- tion, and were treated with cauterization and paper patch and tympanoplasty surgery, respectively. Two patients (2.6%) had sarcoidosis.

## Discussion

In this study, we found that combined therapy with intratympanic and systemic steroids is more effective than systemic steroids alone in the treatment of poor-prognosis SSNHL. Hearing improvement after treatment was significantly higher in the intervention group than in the control group (75% versus 41.4%, respectively). 

Intratympanic steroids have only minor local morbidities, and their efficacy as first-line therapy without systemic steroid in SSNHL has been shown. A higher concentration of therapeutic agent in the cochlea is associated with greater hearing recovery in animal studies (1). According to studies in guinea pigs, when medication is administered through the transtympanic route, a much higher concentration of steroids is achieved compared with systemic administration (1). It has been shown in animal models that intratympanic steroids cause no morphologic or functional compromise, although there are some reports of tympanic membrane perforations and otitis media secondary to the perfusion process in human studies (14). It has also been shown that the chance of salvaging hearing decreases if the time interval between the insult and the administration of intratympanic steroid therapy after oral steroid failure increases. If intratympanic steroids are to be used, therefore, they should be used as soon as possible after it becomes clear that systemic steroids are not effective, preferably within 2 weeks of the original insult (14). To date, few clinical trials have been performed on the combination of intratympanic and steroid therapy. In combination therapy, the patient theoretically benefits from the therapeutic effects both of systemic and local steroids. 

To the best of our knowledge, studies by Battaglia et al. (1,7), Ahn et al. (1,12), and Arslan et al. (15) are the only randomized clinical trials on combination therapy. Ahn et al. (12) evaluated the therapeutic efficacy of intratympanic dexamethasone injections added to systemic steroids in SSNHL patients, and concluded that in comparison with systemic steroids alone, this therapy did not result in significant improvement in the treatment of ISSNHL. Battaglia et al. (7) evaluated the therapeutic efficacy of adding intratympanic dexamethasone to a high-dose prednisolone taper (HDPT) in the treatment of SSNHL, and suggested that combination therapy offers a higher likelihood of recovery in ISSNHL than HDPT alone. Arslan et al. compared hearing results in ISSNHL patients treated with intratympanic methylprednisolone and systemic steroids against systemic steroids alone and concluded that adding intratympanic methylprednisolone to systemic therapy increases the probability of hearing recovery in ISSNHL patients (15).

The difference between our study and the earlier ones is that we chose a poor prognosis subgroup of SSNHL patients. The reason for performing the study in this subgroup was to investigate the most rigorous treatment as first-line therapy for patients with the poorest prognosis. 

One of the limitations of our study was that our local ethics committee at the department of otolaryngology did not allow us to use an intratympanic injection with empty syringe in the control group to eliminate the placebo effect of injection, and so patients were not blinded to the treatment they received. Second, we did not account for vertigo as a poor prognostic factor in the inclusion criteria. Nevertheless there was no statistical difference in the incidence of vertigo between the two groups (Table 1). 

## Conclusion

This clinical trial showed that the combination of intratympanic and systemic steroids is more effective than systemic steroids alone in the treatment of SSNHL patients with poor prognostic factors. Further studies will be needed to show the efficacy of combination therapy as first-line therapy in all patients with ISSNHL.
